# Mass media campaigns for the promotion of oral health: a scoping review

**DOI:** 10.1186/s12903-022-02212-3

**Published:** 2022-05-14

**Authors:** Eileen Goldberg, Joerg Eberhard, Adrian Bauman, Ben J. Smith

**Affiliations:** 1 Sax Institute, Glebe, NSW 2037 Australia; 2 Sydney School of Dentistry, The University of Sydney, Sydney, NSW 2006 Australia; 3 Charles Perkins Centre, The University of Sydney, Sydney, NSW 2006 Australia; 4 Westmead Applied Research Centre, Westmead Hospital, Westmead, NSW 2145 Australia; 5 Prevention Research Collaboration, Sydney School of Public Health, Lev 6, Charles Perkins Centre (D17), The University of Sydney, Sydney, NSW 2006 Australia

**Keywords:** Mass media campaign, Oral health, Systematic review, Program evaluation

## Abstract

**Background:**

Oral diseases are highly prevalent globally and are largely preventable. Individual and group-based education strategies have been dominant in oral health promotion efforts. Population-wide mass media campaigns have a potentially valuable role in improving oral health behaviours and related determinants. This review synthesises evidence from evaluations of these campaigns.

**Methods:**

A systematic search of major databases was undertaken to identify peer-reviewed articles reporting the evaluation of mass reach (non-interpersonal) communication strategies to address common forms of oral disease (i.e., dental caries, periodontitis, gingivitis). Studies using all types of quantitative design, published in English between 1970 and 2020 were included. Data concerning campaign objectives, content, evaluation methods and findings were extracted.

**Results:**

Eighteen studies were included from the 499 identified through searching, reporting the findings of 11 campaign evaluations. Two of these used controlled quasi-experimental designs, with the remainder using pre- and post-test (N = 5) or post-test only designs (N = 4). Message recall, as a measure of exposure, was reported in eight campaigns with short-term (≤ 8 weeks) recall ranging from 30 to 97%. Eight studies examined impacts upon oral health knowledge, with four of the five measuring this at baseline and follow-up reporting improvements. From the eight studies measuring oral health behaviours or use of preventative services, six that compared baseline and follow-up reported improvements (N = 2 in children, N = 4 in adults).

**Conclusion:**

There are relatively few studies reporting the evaluation of mass media campaigns to promote oral health at the population level. Further, there is limited application of best-practice methods in campaign development, implementation and evaluation in this field. The available findings indicate promise in terms of achieving campaign recall and short-term improvements in oral health knowledge and behaviours.

**Supplementary Information:**

The online version contains supplementary material available at 10.1186/s12903-022-02212-3.

## Background

Oral health refers to a level of health of the mouth, gums, teeth, jaw and related tissues that allows a person to eat, speak, and socialise without the impediments of disease, discomfort, or embarrassment [1], facilitating comfortable participation in everyday activities at school, at work, at home and other settings [2]. Oral disease incorporates a range of disorders that include dental caries, gum (periodontal) disease, tooth loss, embedded and impacted teeth, and diseases of salivary glands, lips, oral mucosa and tongue [3]. The most recent global burden of disease study estimates that oral diseases are highly prevalent worldwide, affecting 3.5 billion of the world’s population [4]. Of these the most common conditions are untreated caries in permanent teeth (29.4%, or 2.3 billion people), severe periodontitis (9.8%, or 796 million), untreated caries in deciduous teeth (7.8%, or 532 million) and total tooth loss (3.3%, or 267 million) [4]. The World Health Organization (WHO) has recognised that oral diseases constitute a major public health problem given their high prevalence and incidence in all regions of the world, the interrelationship between oral health and overall health, and the fact that poor and disadvantaged population groups carry the greatest burden of disease [5].

The WHO’s approach to oral health promotion concentrates on reducing intermediate modifiable risk factors related to lifestyle, common to many non-communicable diseases (NCDs) (e.g., cardiovascular disease, diabetes, chronic obstructive pulmonary disease), in addition to promoting the use of fluoride, oral health services and oral hygiene practices [5]. The role of diet (particularly sugar consumption), tobacco use and excessive alcohol consumption are emphasised as important risk factors for oral disease, while the value of oral examination in detecting signs of other conditions in the body are highlighted.

While recommendations to tackle the burden of oral disease have emphasised the need for population-wide approaches, the focus of much oral health promotion research has been upon education and behaviour change strategies delivered to patients in dental care, and to community members in selected settings, particularly schools. Strategies tested in clinical environments have included delivery of advice, motivational interviewing, handouts, pamphlets, mailed postcards, and video demonstrations [6, 7]. In recent years there has been an increase in trials of mHealth strategies in dental care, which in most cases have been via text messaging, and in some instances mobile phone applications [8]. Outside of the clinical context, studies have investigated the efficacy of oral health education strategies for selected population groups, including children, adolescents, women in pregnancy, and Indigenous communities, using methods such as classroom presentations, booklets, leaflets, audiovisual aids and financial incentives [9, 10]. In many of these studies significant effects have been shown upon markers of oral health status, particularly dental caries and gingivitis, as well as oral hygiene behaviours (e.g., tooth brushing, flossing) and related knowledge and attitudes.

The important role that health promotion and disease prevention plays in the oral health care system is widely recognised, but there have been calls for this to be rebalanced to achieve greater public health impact [11]. This will require less reliance on downstream individual and group-based interventions, and greater investment in mid-stream actions to influence health behaviours at the population level, and upstream strategies (e.g., taxes, reimbursements) to address the social determinants of oral health [12]. At the mid-stream level, mass media campaigns (MMCs), which are defined as purposive, population-focused and persuasive communications campaigns to improve health, may have a valuable role to play. MMCs aim to increase whole-of-community understanding, shape an agenda for change, and often present a range of potential change options or information-seeking steps that could lead to health-enhancing behaviours. The evidence concerning the impacts of MMCs using television, radio, newspaper and other electronic and print media shows that these can have significant effects upon major public health risk factors, including tobacco use, sedentary behaviour, sexual health practices, sun protection behaviours, cancer screening, and road safety behaviours [13–15]. The expansion of digital communication options over the past 25 years, including web advertising, online video, social media, and “blast emails”, has increased the range of tools that campaign developers can draw upon, and these are showing promising impact in multiple areas of behaviour change [16, 17]. Further, there is encouraging, albeit limited, evidence that MMCs can contribute to the development of public health policies, as reported in relation to clear air legislation and tobacco sales regulations [16].

For oral health, MMCs can be used to target preventive health behaviours, improve screening or encourage the use of dental services. They may also be applied in advocacy efforts to raise public awareness and support for policy initiatives to improve oral health, such as water fluoridisation and subsidisation of dental services for priority population groups. It is notable, however, that there has been limited attention to MMCs in previous reviews of the evidence concerning oral health promotion strategies. The purpose of this scoping review is to describe the objectives, design and evaluation methods of oral health MMCs, and to report current evidence of their effectiveness, strengths and limitations.

## Methods

This scoping review was registered at the Research Registry (ID: reviewregistry1288). A systematic search for articles was conducted to identify studies reporting the evaluation of MMCs for the promotion of oral health and/or the prevention of common forms of oral disease (i.e., dental caries, periodontitis, gingivitis). Articles eligible for inclusion were those examining the impact of interventions that disseminated oral health messages to population groups using mass-reach (non-interpersonal) methods, including electronic, digital and/or print media. To be included, articles were required to be published in English between January 1970 and December 2020. Exclusion criteria were: use of mass media for commercial marketing of dental products or services; reports of content analysis of oral health messages in the mass media; qualitative studies; and, non peer-reviewed ‘grey literature’ publications.

**Fig. 1:**
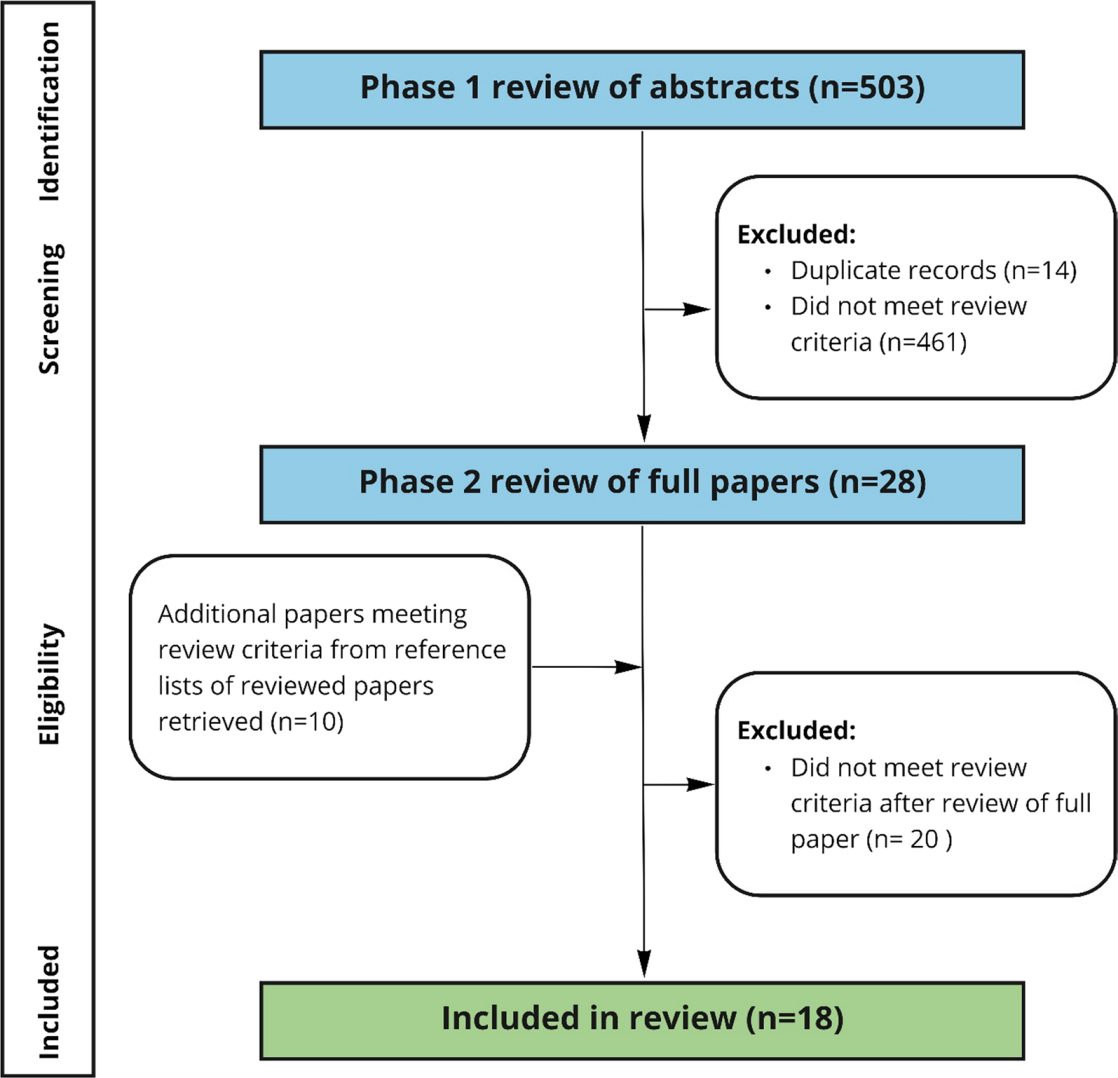
Flow diagram showing numbers of articles identified, screened, and included

The literature searching strategy is shown in Additional file 1: Figs. S1 and 2. One author (EG) conducted the searches of the OVID Medline and SCOPUS electronic databases, removed duplicates (N = 14), and screened the titles and abstracts of 489 articles against the inclusion criteria. This process yielded 28 abstracts (Fig. 1). The abstracts of a sample of 10% of all articles identified (excluding duplicates) were reviewed separately by a second author (AB) against the inclusion criteria. Reviewer agreement was found to be 86%. Papers were assessed against the inclusion criteria and the reference lists were checked for additional studies not identified via the systematic search. During this process, a further 10 papers were identified, located and assessed for inclusion. Of the 38 full articles assessed, 20 articles did not meet the review inclusion criteria, leaving 18 published papers in the final review.

### Extraction and synthesis of evaluations

One author (EG) extracted information from the included articles to identify the country of origin and implementation period of the MMC, geographic scale of the campaign, target audience, messages (theme/brand), media channels used, other campaign elements, evaluation methods, and study findings. The FLOWPROOF framework for the appraisal of mass media campaigns was used to analyse the extracted information [18], as it encompasses the best practice elements of campaign development, delivery and evaluation. The components of the FLOWPROOF framework are shown in the adjacent Box 1.

**Box 1:** FLOWPROOF framework for the appraisal of mass media campaigns

**F** ormative research/evaluation	Assessment of needs to be addressed by campaign and pre-testing of campaign elements
**L** ogic model/use of theory	Theoretical or planning framework for design of campaign and/or its evaluation
**O** bjectives	Specification of behavior and non-behavior outcomes and indicators, and target populations
**W** ell-resourced	Financial, human and organisational resources used to run the campaign
**P** rocess evaluation/**R** un the campaign	Reporting of campaign delivery, fidelity, reach to audience, perceptions/satisfaction, contextual influences
**O** n-the-ground support	Ancillary programs and activities in community or selected settings to support campaign
**O** utcomes/impact evaluation	Evaluation of the campaign against objectives and indicators, and design(s) used to determine this
**F** inancial evaluation	Reporting of cost of the campaign and assessment of cost–benefit, cost-effectiveness, or return on investment

## Results

The 18 articles included in this review described 11 campaigns. Table 1 presents the data extracted from these article for each of these campaigns. The campaign locations were widely dispersed, with seven in European nations (Finland (N = 2), Norway, Sweden, Netherlands, Ireland, Scotland), and the remainder in the United States, China, Iran and Australia. Of these, eight were at the national or large regional level, and three were at the city or smaller regional level.

**Table 1:** Methods of and findings of oral health mass media campaign evaluations

Author, campaign year	Scale and location	Target audience	Campaign messages	Media channels	Other campaign elements	Evaluation methods	Sample size, response rate	Campaign exposure	Knowledge, attitude, behaviour change
Bakdash et al. [19, 20]	Statewide campaign Minnesota, USA	Adults (18+ years), who did not visit a dentist at least once per year	Periodontal awareness (using health belief model) Message of TV advertisements: “Keep your teeth…before gum disease has you looking for a place to keep them”	Paid advertisements on TV, radio, billboards, bus-side posters		*Process evaluation* Campaign exposure assessed at 2 month follow-up survey *Impact evaluation* Cross-sectional interviews 2 months post-campaign. Probabilistic, multi-stage cluster sampling used	N = 1000 adults (response rate not given)	79% of respondents reported exposure to TV campaign, and 71% correctly recalled campaign message	*Knowledge* 65% of those recalling the campaign could report causes of tooth loss, vs 56% of those who could not recall (*p* < 0.05) *Behaviours* 10% who recalled the campaign vs 6% who did not recall expressed intention to make more preventive dental visits (NS)
Murtomaa and Masalin [21]	National campaign, Finland	15–50 years	Increase demand for dental services as preventive measure to improve dental health Main message: “Teeth can be kept throughout life”	Paid TV, radio, newspaper and magazine	Information-based mail-campaign aimed at informing dentists about recalling patients preceded the main media campaign Local dental societies arranged free dental visits to coincide with campaign	*Impact evaluation* Pre- and post-test, with baseline data from national survey on dental service utilisation in 1980 [2 years before campaign], and post-campaign survey in 1983 [12 months after campaign]. Probabilistic, multi-stage cluster sampling used	Pre-campaign N = 648, post-campaign study: N = 694 (response rates not given)	No measures of campaign recall	*Behaviour* Higher proportion visited the dentist in the past 12 months (65% vs. 54%, *p* < 0.001) and 24 months (87% vs78%, *p* < 0.001). Proportion whose last visit was for routine examination was slightly higher (40% vs 36%, NS)
Schou [22]	National campaign, Scotland	Children 5–7 years and their mothers	Raise awareness about restricting sugar intake to meal times and regular toothbrushing with fluoride toothpaste Campaign slogan: “Go for Good Teeth”	TV (advertisement featuring ‘Bugs Bunny’ shown during children’s viewing time), and pamphlet insert in magazines	Dental health information package distributed via primary schools (poster, letter to parents, ‘brushing scorecard’ to return to school, reward badge, mirror sticker)	*Formative evaluation* Acceptability and comprehensibility of TV advertisement tested with groups of 5–7 year olds and their parents *Process evaluation* Campaign exposure and satisfaction, assessed by follow-up interviews with mothers and children post-campaign *Impact evaluation*. Post-campaign survey with assessment of association between recall and oral health behaviours immediately and after 2 months. Stratified quota sampling was used	Sample: mothers n = 164; Children n = 164	Prompted recollection of any campaign elements was 77% among mothers and 97% among children; in both groups the scorecard was most often recalled	*Behaviour* 34% of children recalling the campaign increased their tooth brushing, and 35% reported less sugar intake. 64% of children using the tooth brushing score card
Rise and Sogaard, and Sogaard [23, 24]	National campaign, Norway	15+ year olds	Awareness of causes and symptoms of periodontal disease and knowledge of preventive behaviours Theme was “Perio-Year”, the year against periodontal disease”	Paid radio, TV, newspaper and magazines Some unpaid TV, radio and newspaper	One year of periodontal training and information provided to dentists prior to the campaign, so they would reinforce campaign messages during patient dental visits Booklets distributed to grocery stores, pharmacies and dental offices	*Process evaluation* Reach via different media channels assessed in first post-campaign survey, and exposure (recall) measured at all follow-up surveys *Impact evaluation* Pre- and post-test, with follow-up surveys conducted immediately after, and one and three years later. Sampling methods for respondents not described	Survey respondents N = 1100–1200 from baseline (1981), to each follow-up in (1982, 1983, 1985). Sample sizes and response rates not reported	57.7% of participants had prompted recall of seeing information about oral health in mass media immediately after campaign (1982) compared to 47.7% in the year after (1983) and 46.6% 3 years later (1985)	*Knowledge* From 1982 to 1983 knowledge of tooth brushing to prevent gingivitis increased from 31.7% to 37.9%, and of interdental aids 47.3% to 55.3% (*p* < 0.05); no increase observed from 1981 to 1982, or 1983 to 1985 *Behaviours* Prevalence of daily flossing increased from 20.1% to 24.9% from 1982 to 1983 (*p* < 0.05); NS in the other years, or in the prevalence of daily use of tooth picks
Bian et al. [25] and Dai et al. [26]	National campaign, China; urban and rural communities	Whole population	“Love Teeth Day” with main message of toothbrushing and using fluoridated toothpaste. Themes added and modified each year	Paid TV, radio, newspaper, to publicise events every year from 1989 to 2010	Lectures/symposia/knowledge contests, posters, pamphlets, cartoon strips and slides Face-to-face consultations in public spaces. Oral health education and therapeutic work for oral disease with mobile dental equipment in the community and at schools	*Process evaluation* Delivery and reach assessed by reports from local program organisers in two cities and two counties in each province (1989–1992) *Impact evaluation* Annual post-campaign questionnaires sent out to public by local program organizers in cities and counties where process evaluation undertaken, 1 month after campaign. Population sampling methods not described	Between 321 and 764 adults each year from 1989 to 1992 (response rates not given)	No measures of campaign recall	*Knowledge* Proportion of correct answers to oral health knowledge questions rose from 37% in 1989 to 77.7% in 1992 *Behaviour* Between 1989 and 1992: tooth brushing twice per day rose from 50% to 69.3%; use of fluoridated toothpaste rose from 13.7% to 43.6%; use of qualified toothbrushes increased from 49.1% to 86%; levels of never visiting dentists were 17.4% and 18.3%, respectively
Koelen et al. and van der Sanden-Stoelinga et al. [27, 28]	National campaign, The Netherlands	Parents of children aged 9–18 months	Awareness of prevention of nursing caries in babies Campaign slogan: "Bottle it up—take a cup! From 9 months onwards"	Paid TV advertisements Unpaid coverage in dental health journals, day-care/playgroup journals, magazines for parents of young children, newspapers, radio and TV	Materials (posters brochures, colouring picture and letter for parents) distributed to child health clinics, municipal public health services, dental services, health shops, children’s hospitals, day-care/ playgroups	*Formative evaluation* Assessment of salience, clarity, and comprehensibility of campaign brochure and poster by interviews with samples of parents at child health clinics *Process evaluation* Reach to intermediary child health clinics, satisfaction with materials and contextual influences assessed by follow-up survey; reach to parents by different resources assessed by follow-up survey *Impact evaluation* Pre-post survey of child health clinics and parents at baseline and 18 months post campaign. Random sample of clinics selected. Sampling methods for parents not described	At baseline N = 128 parent using child health clinics (response rate = 94%), and at follow-up N = 98 (response rate = 98%)	46% of parents reported seeing the poster, 23% receiving information, and 10% had been given a brochure 50% knew the campaign slogan after the campaign	*Knowledge* After campaign 78% of parents had heard of nursing caries, vs 60% at baseline (*p* < 0.05) *Behaviour* Parents reported using bottle less after the campaign compared (88% vs 64%; *p* < 0.001). Higher proportion of parents switched from bottle to drinking cup before 12 months of age (88% vs 72% before campaign (NS)
Friel et al. [29]	National campaign, Ireland	School children 7–12 years	Oral hygiene; frequency and duration of tooth brushing, amount and type of toothpaste, when to replace toothbrush Advertisements used ‘Smile of the Year’ competition to promote oral hygiene knowledge	Paid TV advertising delivered via a children’s TV program over 6 weeks	Primary school dental nurse-led health education intervention	*Process evaluation* Campaign exposure assessed at follow-up survey *Impact evaluation* Quasi- experimental controlled pre- and post test design in 32 schools, with follow-up at 8 weeks after intervention. Schools selected using stratified random sampling. Sampling methods for students not described	At baseline: N = 769 experimental and N = 765 control (response rates not given). At follow-up: N = 743 experimental and N = 659 control	62.9% of children reported exposure to the TV campaign	*Knowledge* 75.9% of 11–12 year olds exposed to nurse education plus TVC had knowledge of fluoride toothpaste, vs 65.5% exposed to nurse education only (*p* < 0.01); no difference in knowledge about sugary foods *Behaviours* 7–8 year olds exposed to nurse education plus TVC compared with nurse education only had higher levels of brushing twice per day (78% vs 68.4%, *p* < 0.05), brushing for 3 min (42.5% vs 25%, *p* < 0.01) and using right amount of toothpaste (49.0% vs 35.1%, *p* < 0.01) 11–12 year olds those exposed to nurse education plus TVC had higher levels brushing for 3 min (54.1% vs 47.9%, *p* < 0.01) and using the right amount of toothpaste (64.0% vs 43.6%, *p* < 0.01)
Martensson et al. [30, 31]	National campaign, Sweden	50–75 years	Knowledge of periodontitis	Paid programme on TV Unpaid newspaper, radio and TV coverage	Brochures for dental clinics	*Process evaluation* Exposure assessed at follow-up survey *Impact evaluation* Pre- and post questionnaires of cohort, with follow-up after 6 months. Probabilistic sampling of parents within a panel	N = 630 completed baselined questionnaire (response rate = 70%), with 88.6% of these completing 6 month follow-up	No measures of campaign recall	*Knowledge* Increased knowledge of mobile teeth as a symptom of poor dental health (65% vs 57%, *p* < 0.01) and role of careful dental hygiene (73% vs 65%, *p* = 0.001)
Tolvanen et al. [32, 33]	Regional campaign, Finland	School children and their carers	Increase daily toothbrushing frequency Campaign slogan: “Once a day is not enough”	Public relations activity to generate unpaid TV coverage	Prior to campaign *c*hildren received oral health education in school, and in stores, at fairs	*Impact evaluation* Controlled pre- and post-study comparing both children and parents in Pori with those in the municipality of Rauma after 1 and 3.5 years. All children in the designated school years were selected in each town	Baseline (2001): Pori (intervention group) children N = 1649 (response rate = 97.5%), carers N = 1527 (response rate = 90.3%); Rauma (control group) children N = 734 (response rate = 91.0%), carers N = 693 (response rate = 85.8%) Follow-up (2005): Pori (intervention group) children N = 1598 (response rate = 96.3%), carers N = 1292 (response rate = 77.9%); Rauma (control group) children N = 749 (response rate = 90.6%), carers N = 523 (response rate = 63.2%)	No measures of campaign recall	*Knowledge* Trend to improved knowledge of oral health behaviours among children and carers in 2005, but NS differences between groups *Attitudes* Improvements in attitudes towards oral health tended to be greater in children in the control region in 2005, but NS differences NS difference in attitude among carers *Behaviours* In 2005 all children showed behaviour improvement, but intervention town children had lower consumption of sugary snacks, sports drinks and xylitol products, and smoking prevalence. Improvements in behaviours were not greater among carers in intervention towns
Sivaneswaran et al. [34]	Rural town, Australia	Adults 18 years and over	Promoting benefits of water fluoridation ahead of a plebiscite about this policy	Paid newspaper advertisement. Unpaid newspaper, radio and TV	Posters, pamphlets, ‘how to vote’ cards. Lobbying to mobilise the community; children as advocates	*Impact evaluation* Post-campaign plebiscite of all on electoral roll to measure support for water fluoridation	N = 4,539 (response rate = 86%)	No measures of campaign recall	*Attitudes* 55.8% of voters agreed with fluoridation of water
Gholami et al. [35, 36]	National campaign, Iran	18–50 years	Knowledge of oral health and periodontal disease	Public service television advertisement delivered via a video animation clip		*Process evaluation* Campaign exposure measured at time of follow-up survey. At follow-up also measured satisfaction with campaign (appeal, usefulness, relevance, recommended to others) *Impact evaluation* Survey via interview at baseline and follow-up of cohort immediately following campaign and 3 months later. Probabilistic, multi-stage cluster sampling used	At baseline N = 791 adults (response rate not given); follow-up of 68.6% immediately post-campaign and 37.2% after 3 months	30% aware of campaign at immediate follow-up	*Knowledge* Post-campaign knowledge of plaque and gum disease improved more in those recalling the campaign compared with those who did not (52.9% vs 39.1%); mean knowledge score of 0.61 in the exposed vs 0.29 in the unexposed (*p* < 0.01) At 3 months knowledge scores did not differ between the exposed and unexposed

NS, non-significant; TVC, television commercial

### Campaign development

The vast majority of the evaluations [10 out of 11] reported formative needs assessment data as the rationale for the campaigns conducted. In most instances this was evidence of the prevalence of poor dental health (e.g., caries, decayed missing and filled teeth, periodontitis) from population surveys [22, 25, 27, 29, 30, 35]. In two evaluations [23, 32] the lack of impact of previous oral health promotion strategies upon behaviours and indicators of oral health were cited as the basis for the campaigns.

All of the campaigns stated clear objectives, with four addressing periodontal awareness and knowledge [19, 23, 30, 35], six targeting self-care dental preventive behaviours (tooth brushing, toothpaste use, flossing, reducing sugar intake, use of infant drinking cups) [22, 23, 25, 27, 29, 32], and two promoting use of dental health services [21, 35]. One campaign was undertaken to persuade adults in a regional community to vote in favour of water fluoridation [34]. Most of the MMCs did not have well defined target audiences. In two campaigns it was noted that campaign messages were directed to a whole population [25, 32], while five campaigns targeted adults [19, 23, 30, 34, 35], and two targeted children [22, 29]. One campaign focused on parents of infants [27], and another targeted ‘at high-risk’, lower socio-economic groups [26].

While none of the campaign evaluations presented a comprehensive logic model, three cited a theory or model of change as the basis for their campaign design [19, 23, 27]. Both the “Bottle it up” nursing caries prevention campaign in the Netherlands [27] and the “Perio-year” campaign in the Norway [23] recognised the importance of social influences, in addition to individual knowledge and attitudes, for promoting health behaviours. Consequently, each included strategies to engage intermediaries (e.g., dentists, child health clinics) through awareness raising and resource provision in order to improve the education and support given to the target audiences. In the “Keep your teeth…” campaign in the state of Minnesota in the United States [19], the Health Belief Model was applied in the design of messages and materials.

### Campaign delivery, on-the-ground support and resourcing

All except two [32, 35] of the MMCs used paid advertising to reach target audiences via the mass media. Six of the campaigns made use of unpaid media and/or public service announcements [23, 27, 30, 32, 34, 35]. There was only one oral health campaign that was implemented over multiple waves [25], involving a different theme every year for over two decades.

In addition to mass media, in four campaigns information and resources were provided to dental professionals to boost on-the-ground support for the oral health messages through use of these materials in their interactions with the target groups [21, 23, 27, 30]. For instance, the “Bottle it up” campaign in the Netherlands included information and resources for nurse-practitioners in child health clinics prior to the broadcast of a television advertisement targeting parents of babies and young children [27]. In another campaign in Finland, local dental societies offered free dental consultations to coincide with the campaign [21]. In three campaigns where key messages were targeted at children and/or their parents and caregivers, the MMCs were accompanied by education interventions delivered via childcare centres and local primary schools [22, 27, 29].

Little information was available in any of the evaluations concerning finance, personnel and other resources required to deliver the MMCs and associated activities. The article reporting on the water fluoridation advocacy campaign in Australia [34] was the only instance where this detail was given, in which it was stated that the cost of printing posters, “how to vote cards,” and media advertisements was approximately AUS$1,000 (in 2004).

### Campaign evaluation methods

Formative evaluation, in the form of pre-testing of campaign messages and/or resources prior to their implementation, was reported in two campaigns [22, 28]. In the “Go for good teeth” campaign in Scotland [22] the television advertisements were pre-tested with groups of 5–7 years and their parents (number not given) to assess their acceptability and clarity. The development of the “Bottle it up” campaign in the Netherlands [28] included interviews with parents attending child health clinics to pre-test the campaign posters (with 100 parents) and brochures (with 40 parents), in regard to their salience, clarity, and comprehensibility.

Process evaluation was reported in eight campaigns [19, 22, 23, 25, 27, 29, 30, 35]. While it was common for elements of campaign delivery (e.g., frequency, duration, scale) to be stated, there was only one study which reported the methods used to record this information. This was described in the “Love Teeth Day” oral health awareness campaigns in southern China [25], in which program organizers in two cities and two counties in each province reported on the establishment of consultation stations and dissemination of written materials.

The methods used to evaluate campaign reach were stated in three studies [23, 25, 27]. In the “Bottle it up” campaign in the Netherlands these data were collected by means of follow-up surveys of target groups [27], which comprised Child Health Clinic staff and parents. In the “Love Teeth Day” campaign in China, the number of people reached was documented by program organisers in cities and counties [25], while in the “Perio-Year” campaign in Norway reach via different media channels was measured by respondent self-report at the follow-up surveys [23].

Campaign exposure was the most common form of process evaluation, which was reported in six campaigns [19, 22, 23, 29, 30, 35]. In all cases, this was measured by asking questions within follow-up surveys to elicit campaign message recall.

Three of the evaluations incorporated an assessment of satisfaction with campaign messages and/or materials [22, 27, 35]. In the national oral health and periodontal disease campaign in Iran, this was undertaken by inclusion of questions about the appeal, value and relevance of the campaign content in follow-up surveys [35], while in the national “Go for good teeth” campaign in Scotland satisfaction was assessed by asking those who could recalled messages whether they considered these to be likeable [22]. In the “Bottle it up” campaign in the Netherlands follow-up surveys with intermediaries (public health, child health, and dental health staff) were used to determine the extent to which they considered the posters that were disseminated to be clear, eye-catching and realistic [27].

The evaluation of the infant feeding campaign in the Netherlands was the only instance where there was examination of contextual factors which affected the implementation process. This was undertaken by follow-up interviews with the public health and childcare intermediaries [27].

In terms of the evaluation of campaign impacts, two of the studies used a quasi-experimental, controlled pre- and post-test design [29, 32]. In the oral hygiene campaign in Ireland, children were followed up after 8 weeks at control and intervention sites [29], while in the toothbrushing campaign undertaken in Finland there was follow-up of parents and children after both 1 year and 3.5 years [32].

Five of the studies assessed campaign impacts using a pre- and post-test design [21, 23, 27, 30, 35]. In two of these, cohorts underwent assessment at baseline and follow-up, which was after 3 months in one study [35] and 6 months in the other [30]. Two studies recruited independent samples at the pre-and post-test measurement points, with one of these undertaking follow-up after 1 year [21] and the other at multiple time-points (1, 2 and 3 years) [23]. In one study, follow-up was conducted after 18 months and included a cohort measured at baseline as well as newly recruited participants [27].

A post-test only design was used for impact evaluation in four of the studies [19, 22, 25, 34]. The evaluation of the long-term national campaign in China was notable because follow-up was conducted in every year of the campaign over 20 years [25]. In other studies follow-up was carried out immediately after the campaign [34], or 2 months later [19], while in one study follow-up was conducted at both of these timepoints [22].

None of the campaigns included an economic evaluation to assess cost–benefit, cost effectiveness, or return on investment from the oral health MMCs.

### Effects on awareness, knowledge, attitudes and behaviours

In the five evaluations that included measures of campaign awareness at up to 2 months, four reported levels of media and/or message recall among adults that ranged from 30–79% [19, 22, 23, 35] and two reported message recall levels among children ranging from 63–97% [22, 29]. The highest levels of awareness were reported in the study that collected measures from relatively small, quota samples of adults and children [22]. A further study reported awareness among adults at 18 months follow-up, which was reported to be 50% in the small sample measured [27].

Impacts upon oral health knowledge were reported in eight studies, with measures that examined understanding of risk factors and symptoms of poor oral health (e.g., nursing caries, mobile teeth, plaque), and/or related prevention behaviours (e.g., tooth brushing, use of inter-dental aids). The evaluation of the “Once a day is not enough” campaign in regional Finland was the only instance where changes in knowledge were compared between an intervention and control group and, while this found a trend towards improved knowledge of oral health behaviours after 3.5 years in the campaign region, this was not significantly higher than in the control region [32].

Four uncontrolled studies showed significant improvements in measures of oral health knowledge, including those investigating change after 6 months [30], 18 months [27] and 3 years [25]. In the “Perio-Year” campaign in Norway there was no increase in oral health knowledge at the immediate post-campaign measurement point, whereas knowledge improvement was found between the 1 and 2 year follow-up intervals [23]. In three further studies there was investigation of whether those reporting campaign exposure at follow-up had higher levels of oral health knowledge than the unexposed. Each of these reported a significant association between campaign exposure and oral health knowledge, with follow-ups between 2 and 3 months in all cases [19, 29, 35].

Only two studies investigated changes in oral health attitudes. In the quasi-experimental campaign evaluation undertaken in regional Finland there was no improvement found in attitudes towards oral health among parents or children in the intervention town after 3.5 years [32]. In the other study, support for water fluoridation was found to have a prevalence of 55.8% in a post-campaign plebiscite in a rural Australian town [34], however the baseline level of support was not measured before the campaign.

Eight studies reported impacts of campaigns upon oral health behaviours, which included toothbrushing, use of fluoride toothpaste, dental flossing, consumption of sugary foods and drinks, smoking, use of infant feeding bottles, and use of dental service. Two of the controlled quasi-experimental studies found improvements in oral health behaviours among children who were exposed to campaign interventions. In the study conducted in Ireland, at 8 weeks’ follow-up children aged 7–8 years in the campaign intervention group had greater improvements than controls in toothbrushing frequency, while both 7–8 year olds and 11–12 year olds in the intervention group had greater increases in toothbrushing duration and appropriate use of toothpaste [29]. Follow-up after 3.5 years in the evaluation of the oral health campaign in regional Finland found lower levels of sugary snack, sports drink and xylitol consumption, and lower smoking prevalence among children in the intervention city compared with the control city, but no greater improvements in oral health behaviours among parents [32].

The studies using pre-and post-test designs reported improvements in selected behavioural outcomes: in the campaign addressing nursing caries in the Netherlands there was a reduction in infant bottle feeding after 18 months, but not higher adherence to recommendations for switching from bottles to drinking cups [27]; follow-up at 12 and 24 months in the national oral health campaign in Finland found an increase in visits to dentists, but not in attendance for general dental examinations [30]; and, in the campaign in Norway which promoted the use of interdental aids there was found to be an increase in levels of flossing between the immediate post-campaign and 12 month follow-ups, but no improvements were reported in other outcomes [23]. In the serial post-test surveys conducted following the annual campaigns in China there was a marked improvement in twice daily toothbrushing and use of recommended toothbrushes and fluoride toothpaste, over a three year period, but little change in the prevalence of dental visits [25]. In the other two studies that assessed impact using post-test designs, one did not find differences in preventive dental visits between those who recalled and did not recall the campaign [19], while the other reported improvements in oral health behaviours among children who recalled the campaign (but did not compare these with outcomes in the non-recallers [22]).

## Discussion

This is the first synthesis of peer-reviewed studies concerning the delivery and impact of oral health MMCs implemented over a 50-year period. Although reviews have been conducted of health education programs for oral health [6, 9, 10] there has not been a structured assessment of those using mass-reach media channels. There was wide variation in the evaluation and reporting of these interventions, which may reflect a limited adoption of the planning frameworks and models used in MMCs conducted for other health-risk behaviours [15, 16, 18, 37], as well as the under-developed status of research and practice in this area. Notwithstanding these limitations, the findings indicate potential for oral health campaigns to achieve good levels of population engagement, and to influence knowledge and behaviours across diverse oral health topics.

All of the studies included in this review used traditional media channels. Television was the most frequently adopted mass-reach strategy, and some MMCs used combinations of radio, print, billboards and bus-side advertisements, supported by public relations strategies. The fact that none of the campaigns were conducted within the past decade may account for the absence of online and social media methods of delivery, which are now widely used communication channels within public health campaigns given their potential reach and relatively low cost [16]. Only one of the included studies reported a systematic method of recording the delivery of campaign components, and none appeared to adopt commonly used metrics of mass media reach (e.g., gross ratings points). These gaps in standard monitoring practices suggest a lack of attention to campaign targeting, and perhaps limited resources and/or expertise for campaign evaluation.

The messages delivered in campaigns aligned with the recommendations of leading dental health agencies, including the adoption of oral hygiene behaviours (e.g., tooth brushing, use of fluoride toothpaste), reduction in sugary food and drinks, appropriate bottle feeding of infants, and regular use of dental services [38, 39]. One campaign was distinguished by its focus on advocating for public support of water fluoridation, rather than a personal behaviour. The breadth of issues addressed across the MMCs highlights the scope for public health interventions in this field, as well as the opportunity to focus on well-defined behaviours, which is a factor that is likely to improve campaign effectiveness [40]. However, only two of the studies reported preliminary formative evaluation to guide the development of messages and design of media content and resources, which is recognised as a standard element of good practice in MMCs [37, 41]. There was also an apparent lack of use of best practice logic models that propose a roadmap linking campaign activities to message exposure, knowledge development, attitude formation, intentions and behaviours [37].

In several oral health MMCs, health opinion leaders (e.g., health clinic nurses, dentists and general practitioners) were targeted to reinforce campaign messages. It would benefit future oral public health endeavours to harness wider community influencers and social networks [16]. There is also scope to move beyond a reliance upon mass media communication alone, towards a social marketing approach that involves the strategic use of an appropriate “intervention mix” [42]. It has been posited that social marketing initiatives can comprise strategies across five domains: altering the environment; regulation and enforcement; provision of services; education; and the communication of information for attitude change. Some of the MMCs reviewed here attended to the provision of services to support behaviour change, and others incorporated education initiatives in the intervention mix [42]. Building upon this, and applying a social ecological analysis of the determinants of oral health, other important targets of change may include public policies that affect costs of sugar sweetened beverages, access to dental services, incentives for primary care practitioners to promote oral health behaviours, and partnerships with agencies and groups that have engagement with priority population groups (e.g., older adults, cultural minorities). A social marketing approach that incorporates actions at these multiple levels will not only increase enablers for behaviour change, but may also achieve more sustained delivery and impact than is possible through MMCs alone. [16, 42].

Building support among policy makers is needed to increase public investment in mass reach oral health promotion campaigns, and researchers can assist by providing evidence concerning the cost effectiveness of different intervention methods and the potential co-benefits that these will have for the prevention of other chronic conditions (e.g., cardiovascular disease, diabetes). None of the MMCs examined in this review provided evidence of cost effectiveness in relation to behaviour change or dental services utilisation, and only one gave details about the cost of intervention components. It should be noted that this has been identified as a common limitation of the evaluation of MMCs across multiple areas of public health [13]. Given the established relationships between oral disease and major conditions like cardiovascular disease and diabetes [5, 43, 44], and the risk factors that oral disease shares with these conditions (e.g., sugar consumption, smoking), there is potential value in modelling the health and economic benefits of MMCs (and other strategies) to promote oral health. There is also an opportunity to communicate these linkages between oral health and NCDs in MMCs; this review did not find any examples where this had been attempted.

Limitations of this review included the exclusion of studies not reported in English, as well as those which were published in the grey (non peer-reviewed) literature. Further, given that the impact measures and follow-up time points in the studies varied considerably, and that four of the 11 campaign evaluations used a post-test design, it was not possible to estimate campaign effect sizes.

It is recommended that future campaigns follow best practice campaign guidelines, including identification of priority population segments, development of program logic models to guide implementation and evaluation, formative pre-testing of messages, use of a mix of strategies that include mobilisation of professional and community influencers, and provision of resources and services to support behaviour change. Building an evidence base to inform policy-makers and campaign managers will require comprehensive evaluation of oral health MMCs at the process and impact levels.

## Conclusion

While there is a substantial body of evidence concerning the impact of narrow reach oral health education strategies in clinical and school settings, this review has found far fewer studies reporting on population-wide oral health MMCs. As is the case with a number of public health programs, these mid-stream interventions can utilise an expansive range of electronic and digital communication channels to extend the reach of oral health promotion efforts. However, there remains a need to better understand the impact that MMCs can have upon oral health knowledge, attitudes and behaviours, and the use of preventive dental services.

## Supplementary Information

**Additional file 1. Figures: **Fig. 1 and Fig. 2 showing the search terms and numbers of articles identified.

## Data Availability

All data generated or analysed during this study are included in this published article.

## Abbreviations

MMC: Mass media campaigns
NCD: Non-communicable diseases
NS: Non-significant
TVC: Television commercial
WHO: World Health Organisation
