# Supplementary material for: Mass media campaigns for the promotion of oral health: a scoping review
Source: BMC Oral Health. 2022 May 14;22:182. doi: 10.1186/s12903-022-02212-3 (PMC9107752; doi:10.1186/s12903-022-02212-3)
Supplement: Supplementary file 1 — Additional file 1. Figures: Fig. 1 and Fig. 2 showing the search terms and numbers of articles identified. [file 12903_2022_2212_MOESM1_ESM.docx]

**Supplementary material**

**Supplementary Figure 1:** Search terms and number of articles identified in OVID Medline database

**No limits: 1970 – December 2020**1. exp Mass Media/ or mass media campaign.mp. n = 42,443
2. social marketing.mp. or exp Social Marketing/ n = 3839
3. dent* n = 526,060
4. oral health.mp. or exp Oral Health/ n = 32,046
5. (carie* adj3 preven*).mp n = 5259
6. exp tooth/ or tooth.mp n = 181,575
7. teeth.mp. n = 107,076
Total n = 898,298

**Supplementary Figure 2.** Search terms and number of articles identified in SCOPUS database

**Search terms combined with ‘AND’**1. exp Mass Media/ or mass media campaign.mp. OR
 social marketing.mp. or exp Social Marketing/ n = 23,871

AND

2. dent* or oral health.mp. or exp Oral Health/ or (carie* adj3 preven*).mp or
 exp TOOTH/ or tooth.mp or teeth.mp. n = 240,003

**Total n = 437**

**Limited to English, Human and Abstract: 1970 – December 2020**1. exp Mass Media/ or mass media campaign.mp. n = 21,497
2. social marketing.mp. or exp Social Marketing/ n = 2670
3. dent* n = 211,377
4. oral health.mp. or exp Oral Health/ n = 20,343
5. (carie* adj3 preven*).mp n = 2813
6. exp tooth/ or tooth.mp n = 95,404
7. teeth.mp. n = 64,104
Total n = 418,208

**Limited to English: 1970 – December 2020**1. campaign OR mass media OR social marketing n = 23,136
2. dent* OR oral health OR carie* prevent* OR
prevent* carie* OR t?th n = 158,618
Total n = 181754

**Search terms combined with ‘AND’**campaign OR mass media OR social marketing AND
dent* OR oral health OR carie* prevent* OR
prevent* carie* OR t?th

**Total n = 66**
